# Anatomy of the sonographic post-cesarean uterus

**DOI:** 10.1007/s00404-021-06074-y

**Published:** 2021-04-23

**Authors:** Ammar Al Naimi, Bartosch Wolnicki, Niki Mouzakiti, Tiana Reinbach, Frank Louwen, Franz Bahlmann

**Affiliations:** 1 Department of Obstetrics and Gynecology, University Hospital, Goethe University Frankfurt am Main, Frankfurt, Hessen Germany; 2 Department of Obstetrics and Gynecology, Buergerhospital - Dr. Senckenberg Foundation, Nibelungenallee 37-41, 60318 Frankfurt am Main, Hessen Germany

**Keywords:** Post-cesarean, Uterus, Niche, Cesarean section, Ultrasound

## Abstract

**Purpose:**

We aim to describe the sonographic uterine anatomy after a cesarean section (CS), test the reproducibility of predefined measurements from the BSUM study, and report the distribution of these measurements.

**Methods:**

This is a descriptive observational study where 200 women with a history of only one CS were recruited 12–24 months postoperatively. A 5–13 MHz micro-convex transvaginal transducer was used for the acquisition of volumetric datasets for evaluating the CS scars. We defined 15 distinct measurements including the residual myometrial thickness (RMT). RMT ratio was calculated as a percentage of RMT to the assumed pre-cesarean anterior uterine wall thickness. A P value below 0.05 is utilized for significant statistical analysis.

**Results:**

Patients were included on average 18.5 months post-cesarean. The uterus was anteflexed in 82.5% and retroflexed in 17.5%. Myometrial defects at the site of CS manifest in two forms, either as a niche or as fibrosis. Patients are classified into four groups: those with isolated niches (45%), combined niches and fibrosis (38.5%), isolated fibrosis (11%), and lacking both (5%). The median RMT ratio for these groups was 63.09, 40.93, 59.84, and 100% with a standard deviation of 16.73, 12.95, 16.59, and 0, respectively. The interclass correlation coefficient (ICC) remained above 0.9 for all distinct measurements among these groups except for those of RMT, where ICC varied between 0.47 and 0.96. The RMT ratio shows a constant ICC at 0.94 regardless of the group.

**Conclusion:**

The post-cesarean uterus is often anteflexed, and a myometrial loss of about 50% is normally expected. The pattern of this loss is in the form of a predominantly sharp-edged and echogenic niche, fibrosis, or a combination of both. The proposed RMT ratio takes these changes into consideration and results in a reproducible quantification. We hypothesize that different adverse outcomes could be attributed to the different scar patterns.

## Introduction

The uterine anatomical changes after a cesarean section (CS) were first described in 1961 when Poidevin used hysterography to show a wedge-shaped CS scar [1]. This finding was then demonstrated with transabdominal ultrasound when Burger examined 48 puerperal women in 1982 and referred to the wedge-shaped defect as an incompletely healed scar [2]. These defects were histologically confirmed as a uterine wall pouch when Morris examined hysterectomy specimens from women who underwent a CS in 1995 [3]. The sonographic finding of this defect is commonly called a ‘niche’, a term first used by Monteagudo in a sonohysterographic study of women with history of CS [4]. Several studies involved with examining the niches for clinical significance show a wide range of prevalence from 19 to 69% [5]. The most common symptoms of the niche are abnormal uterine bleeding and pelvic pain [6]. Published studies show that niches are associated with the risk of placenta accreta spectrum (PAS), placenta previa, and uterine rupture in a subsequent pregnancy [7]. The pathogenesis of niche formation is multifactorial and is not entirely explored, yet a meta-analysis shows that double-layer uterine closure is associated with fewer niches and thicker residual myometrial thickness (RMT) [8].

A standard method of measuring the niche and the post-cesarean uterus did not exist until a Delphi based guideline was published by Jordans et al. in 2019 [9]. Due to the recency of the guidelines, a new distribution of the normal niche measurements does not exist. Moreover, ultrasound technologies have witnessed tremendous developments in recent years and high-frequency matrix three-dimensional probes which considerably improve niche imaging are now widely available. We initiated a study (BSUM study) to examine the post-cesarean uterus equipped with the latest ultrasound technologies and following the most recent guidelines for niche assessment [10].

This paper aims to describe the sonographic uterine anatomy after a CS, test the reproducibility of our predefined measurements, and provide the distribution of these measurements.

## Materials and methods

This is a descriptive observational study reporting measurements from the patients recruited into the BSUM study. Inclusion criteria were a history of only one CS whether elective or unplanned, age above 18, 12–24 months after CS, and gestational age at delivery between 24 + 0 and 42 + 0 weeks. Exclusion criteria were completed family planning, history of two CSs or more, history of vertical hysterotomy, history of additional uterine surgeries, and spoken language other than English or German. Informed consent was provided.

Examinations were performed in a lithotomy position, hips flexed and abducted and with an empty bladder. A 5–13 MHz micro-convex transvaginal transducer, GE RIC6-12-D (Voluson E10, GE Healthcare GmbH, Munich, Germany), was used for the acquisition of three volumetric datasets from each patient where the uterus was completely visualized. The desired planes for evaluating the CS scars were acquired with multiplanar views.

**Fig. 1:**
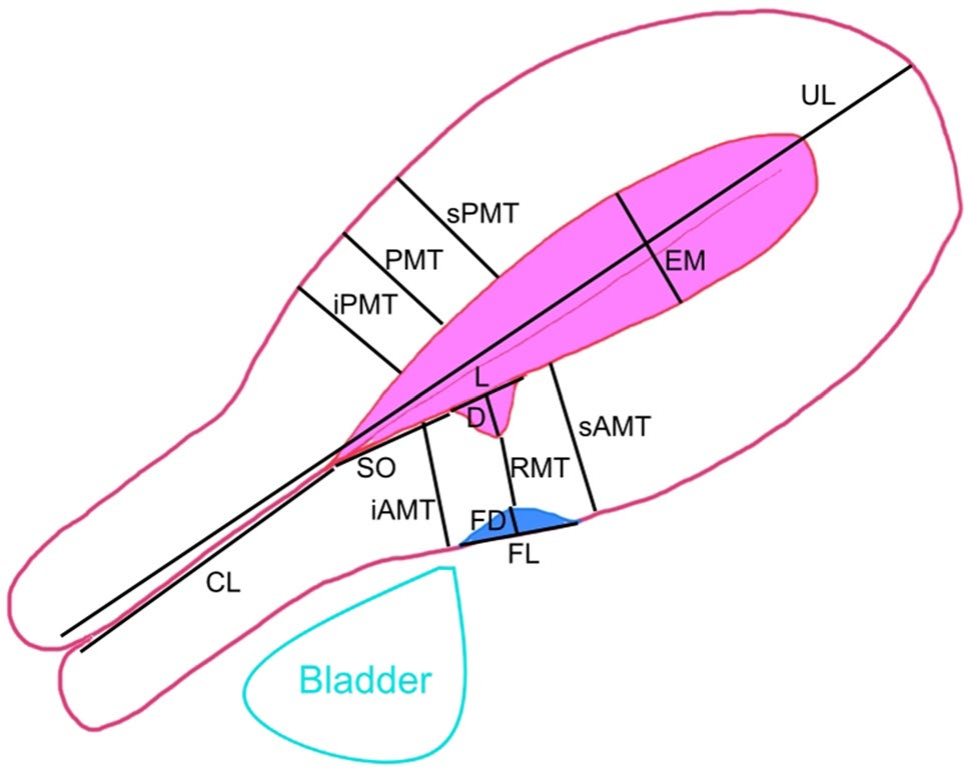
An illustration of the variables measured for evaluating the post-cesarean uterus: uterine length (UL), cervical length (CL), niche length (L), niche depth (D), fibrosis length (FL), fibrosis depth (FD), residual myometrial thickness (RMT), endometrial thickness (EM), scar to internal os distance (SO), anterior myometrial thickness superior (sAMT) and inferior (iAMT) to the scar, and the posterior myometrial thickness opposite the scar (PMT), superior (sPMT), and inferior to it (iPMT)

As per the recommendations of Jordans et al., the length (L) and depth (D) of the main niche were measured in the sagittal plane where they were at their maximum and RMT was measured where it was at its minimum [9]. The uterine length (UL), cervical length (CL), niche length (L), niche depth (D), niche width (W), fibrosis length (FL), fibrosis depth (FD), endometrial thickness (EM), scar to internal os distance (SO), anterior myometrial thickness superior (sAMT) and inferior (iAMT) to the scar, and the posterior myometrial thickness opposite the scar (PMT), superior (sPMT), and inferior to it (iPMT) were measured as per the protocol of our BSUM study [10]. RMT ratio was calculated as a percentage of RMT to the assumed original pre-cesarean anterior uterine wall thickness (summation of RMT, fibrosis depth, and niche depth). RMT ratio = RMT × 100/(RMT + FD + D). These measurements are shown in Fig. 1.

BiAS ver. 11.10 (Ackermann, Goethe University of Frankfurt, Germany) was utilized for performing the statistical analysis using Wilcoxon–Mann–Whitney U test, Pearson’s contingency table, Chi-square test, Spearman’s rank correlation, interclass correlation (ICC), Shapiro–Wilk test, and Passing–Bablok regression. The cut-off point for significance was a *P* value of 0.05.

The study was approved by the Ethical Committee of the Hessen Regional Medical Council, reference number 2019-1138-evBO.

## Results

Two hundred patients with mean age of 37.89 years (95% CI 36.68—37.21) were included in this study between 12 and 24 months after a CS with an average of 18.5 months. The proportion of unplanned CS was 44.6% (95% CI 37.81–51.55) and gestational age at delivery was 37.97 weeks (95% CI 37.51–38.44). The uterine position was anteflexed in 82.5% and retroflexed in 17.5%.

**Fig. 2:**
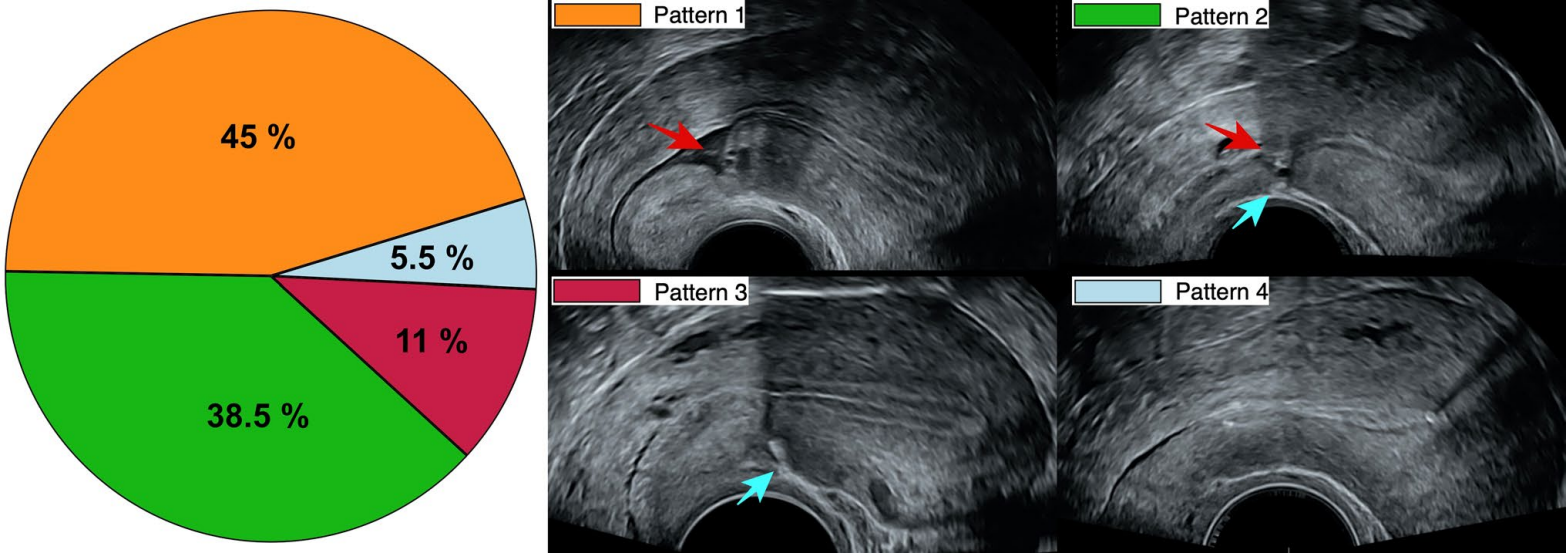
Pie chart representation of the CS scar patterns; pattern (1) with isolated niches, pattern (2) with simultaneous niches and fibrosis, pattern (3) with isolated fibrosis, and pattern (4) lacking myometrial defects. The sonographic appearance of niches and fibrosis is shown with red and blue arrows, respectively

External denting (fibrosis) was identified in 49.5% and a niche was present in 83.5%. There was a minimal, barely significant correlation between fibrosis and niche formation with a Pearson’s contingency coefficient of 0.2 for P value of 0.04. Four patterns of scarring can be identified depending on the manifestation of niches and fibrosis, and the prevalence and sonographic appearance of these patterns are shown in Fig. 2.

The Chi-square test showed no correlation between the position of the uterus and the presence of niches or fibrosis with P values of 0.14 and 0.16, respectively.

**Fig. 3:**
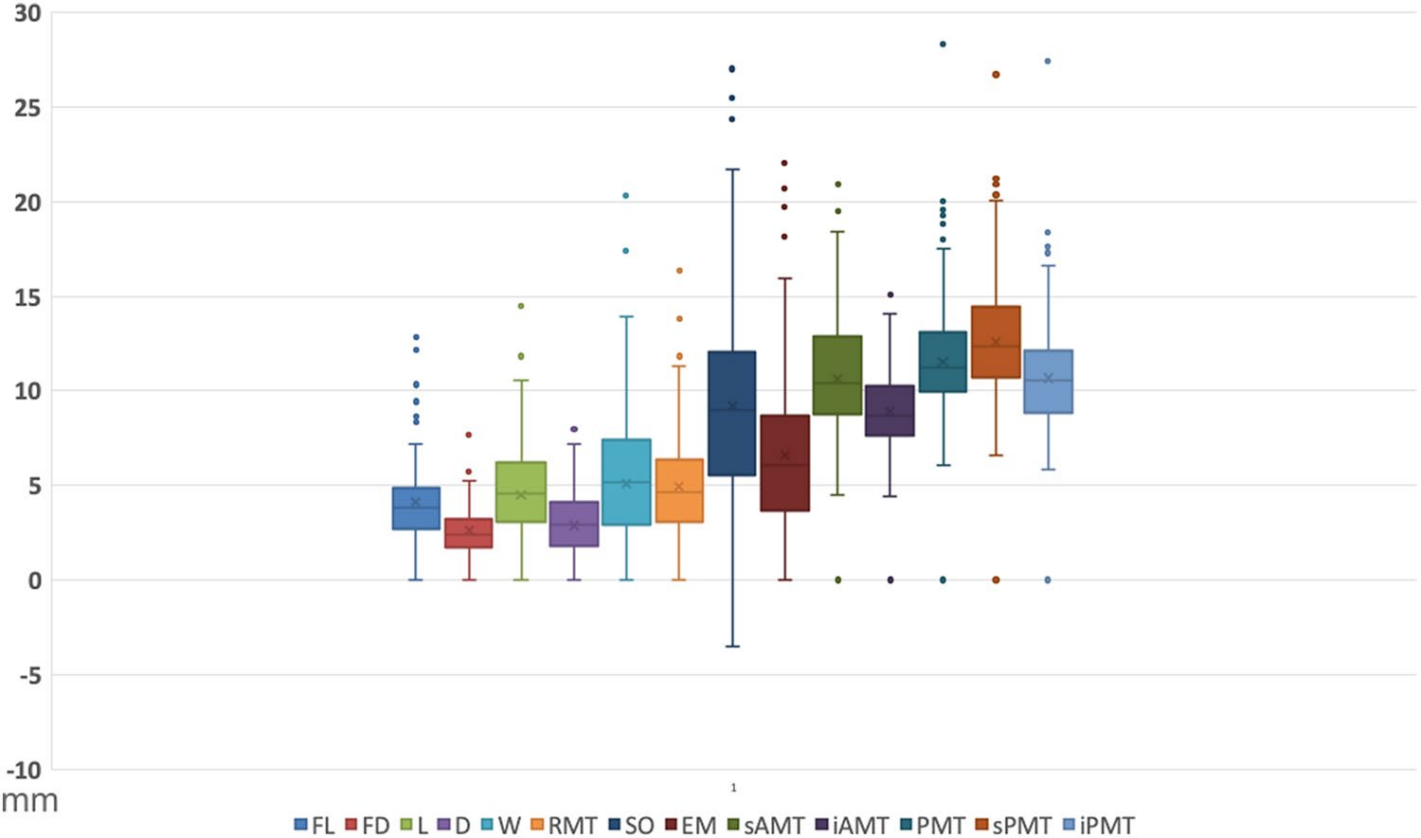
Box plot showing the distribution of fibrosis length (FL), fibrosis depth (FD), niche length (L), niche depth (D), niche width (W), residual myometrial thickness (RMT), scar to internal os distance (SO), endometrial thickness (EM), anterior myometrial thickness superior (sAMT) and inferior (iAMT) to the scar, and the posterior myometrial thickness opposite the scar (PMT), superior (sPMT), and inferior to it (iPMT)

The distribution of all measurements for the recruited patients is shown in a box plot in Fig. 3 and the 95% confidence intervals are listed in Table 1.

**Table 1:** Summary of the ranges, averages, and percentiles for all measurements in the study

Variable	Min	5th percentile	25th percentile	Median	75th percentile	95th percentile	Max	Mean	Range	Standard deviation
FL (mm)	1.43	1.70	2.75	3.83	4.93	8.87	12.87	4.22	11.44	2.20
FD (mm)	0.70	0.99	1.71	2.43	3.22	4.99	7.67	2.62	6.97	1.22
UL (mm)	43.63	51.85	63.18	69.18	74.60	83.94	110.43	68.92	66.80	9.49
CL (mm)	11.07	15.01	18.42	20.90	23.11	25.57	30.20	20.74	19.13	3.21
L (mm)	1.93	2.83	4.01	5.23	6.58	9.92	14.47	5.55	12.54	2.08
D (mm)	1.10	1.50	2.27	3.27	4.31	6.41	8.17	3.45	7.07	1.49
W (mm)	1.73	2.68	3.93	5.67	7.64	11.48	20.30	6.28	18.57	2.94
RMT (mm)	0.87	1.53	3.16	4.71	6.41	9.50	16.37	4.99	15.50	2.50
SO (mm)	− 3.50	2.60	5.73	9.27	12.13	17.61	27.03	9.39	30.53	4.91
EM (mm)	0.73	1.50	3.95	6.13	8.76	13.55	22.03	6.74	21.30	3.85
sAMT (mm)	4.50	6.43	8.80	10.48	12.87	16.27	20.90	10.78	16.40	2.88
iAMT (mm)	4.43	5.95	7.70	8.73	10.30	12.54	15.10	9.02	10.67	2.01
PMT (mm)	6.10	7.68	10.15	11.28	13.17	17.15	28.33	11.71	22.23	2.91
sPMT (mm)	6.60	8.41	10.73	12.37	14.47	18.54	26.73	12.77	20.13	3.13
iPMT (mm)	5.87	6.93	8.95	10.58	12.14	15.93	27.43	10.83	21.56	2.67
RMT ratio (%)	11.93	21.49	38.5	55.5	67.36	100	100	54.42	88.07	20.81

Fibrosis length (FL), fibrosis depth (FD), uterine length (UL), cervical length (CL), niche length (L), niche depth (D), niche width (W), RMT, endometrial thickness (EM), scar to internal os distance (SO), anterior myometrial thickness superior (sAMT) and inferior (iAMT) to the scar, and the posterior myometrial thickness opposite the scar (PMT), superior (sPMT), and inferior to it (iPMT)

The intraobserver reproducibility of all measurements was tested with ICC from three measurement repetitions. The cervical length, depth of fibrosis, and niche length have a high ICC between 0.75 and 0.89. RMT has an ICC of 0.64, whereas the rest of the measurements have an ICC above 0.9 with a significant P value below 0.001. When the measurements were divided into subgroups depending on niche and fibrosis formation, the ICC remained constant for all the measurements except for RMT where ICC varied between 0.47 and 0.96. Moreover, the ICC of the RMT ratio was 0.94 and remained constant regardless of fibrosis or niche formation (Table 2).

**Table 2:** Summary of the interclass correlation test for the predefined measurable variables for the study

Measurement	ICC	95% Confidence interval	MAE	Repeatability
FL	0.93	0.91–0.95	0.57	0.81
FD	0.76	0.69–0.83	0.64	0.91
UL	0.97	0.96–0.97	1.64	2.33
CL	0.87	0.84–0.90	1.17	1.65
L	0.89	0.87–0.92	0.68	0.97
D	0.92	0.90–0.94	0.43	0.6
W	0.94	0.93–0.96	0.69	0.98
RMT	0.64	0.57–0.71	1.71	2.43
SO	0.94	0.93–0.95	1.15	1.63
EM	0.97	0.96–0.97	0.65	0.92
sAMT	0.94	0.93–0.95	0.68	0.96
iAMT	0.90	0.88–0.92	0.63	0.89
PMT	0.94	0.93–0.96	0.66	0.94
sPMT	0.94	0.92–0.95	0.77	1.09
iPMT	0.93	0.92–0.95	0.68	0.96
RMT ratio	0.94	0.93–0.96	4.76	6.74

*ICC* interclass correlation coefficient, *MAE* mean absolute error

The uterine position shows no influence on any of the measurements according to the Wilcoxon–Mann–Whitney U test with an R between 0.1 and 0.23.

**Fig. 4:**
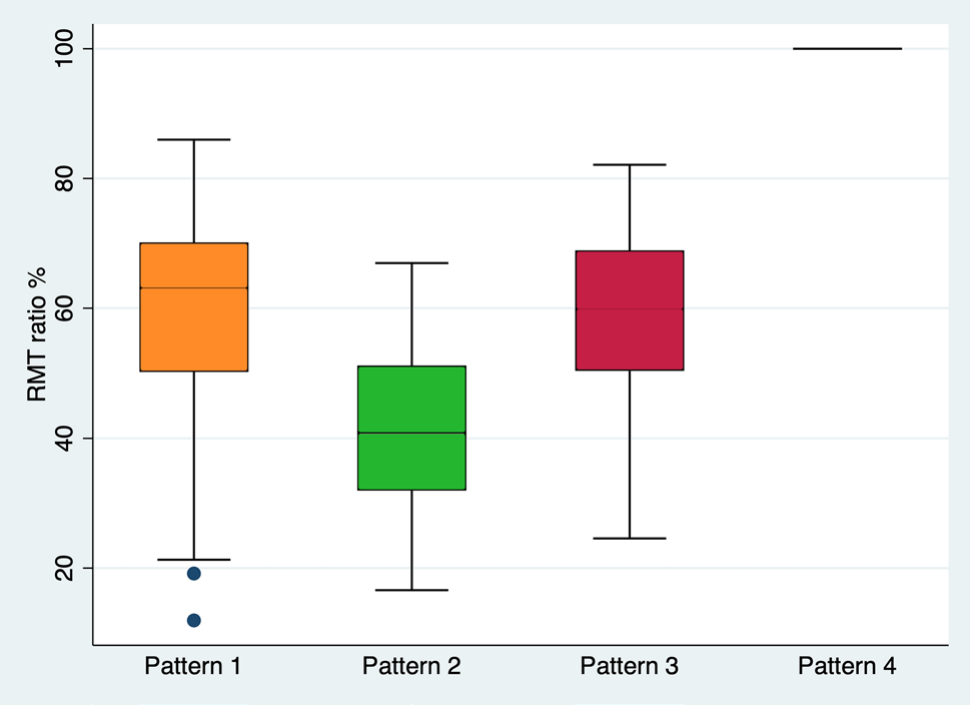
Box plot showing the medians and distribution of RMT ratio for the four patterns of CS scars; pattern 1: isolated niche, pattern 2: simultaneous niche and fibrosis, pattern 3: isolated fibrosis, and pattern 4: lack of both niche and fibrosis

The RMT ratio varies significantly depending on the pattern of niche and fibrosis formation as evident in Fig. 4. The median RMT ratio is 63.09, 40.93, 59.84, and 100% for groups 1, 2, 3, and 4, whereas the standard deviation is 16.73, 12.95, 16.59, and 0, respectively.

The form of the niche was round in 42.1% of cases and sharp-edged in 57.9%, and the niche was also echogenic in 68.2% and hypoechogenic in 31.8%.

## Discussion

The annual percentage of CS deliveries in Germany has doubled from 15.3% in 1991 to 32.9% in 2014 [11], which is a development that mirrors the steady global increase of CS rates in the past 3 decades [12]. These women are faced with several obstetrical complications in subsequent pregnancies. The risk for placenta previa, abruption, and PAS cases increases significantly after a CS [13, 14]. Uterine rupture is the other challenge facing patients undergoing trial of vaginal birth after previous cesarean (VBAC) with an incidence of 0.7% [15]. Rupture can present either as acute symptomatic disruption of both myometrium and the visceral peritoneum under VBAC or as asymptomatic myometrial separation with intact serosa (scar dehiscence) during repeated CS. These risks are the main driving reason for an increase in elective repeated CS in Germany which accounts for one-third of a total of around 180,000 CSs in the country [11].

The changes of the uterine anatomy caused by a CS can be responsible for complications in a subsequent pregnancy, and the ultrasound findings of these changes are believed to be helpful in assessing the probability of obstetrical complications [16, 17]. Most studies were conducted before the methodology for measuring post-cesarean uterine niches was established and standardized by Jordans et al. guidelines [9]. Our BSUM study was initiated with the intention to examine women after CS to recognize candid ultrasound findings for predicting adverse outcomes in a subsequent pregnancy [10].

This paper describes the sonographic uterine anatomy of the first 200 patients included in the BSUM study who were examined on average 18.5 months after the CS. Scar appearance remains consistent 6–9 months after a CS up to the first trimester of a subsequent pregnancy [18]. Therefore, it is safe to assume that the scars of this cohort had reached their full healing potential at the time of recruitment. The uterus is anteflexed in about 50% of women in a normal population [19], but tends to change position into more pronounced anteflexion post-cesarean. While some authors conclude that a CS changes the uterus into a retroflexed position [20], most sonographic studies of post-cesarean uteri show an overall increase of anteflexion up to a prevalence of 80% [21]. This cohort shows similar findings with predominantly anteflexed uteri post-cesarean which could indicate that CS increases uterine anteflexion. This can be attributed to increased tension on the anterior uterine wall from the sutures. It has been suggested that uterine retroflexion is associated with poor CS scar healing due to impaired oxygenation of the scar. Therefore, larger niches are expected in posteriorly flexed uteri post-cesarean [22]. This cohort contradicts such a suggestion and our data showed that retroflexion did not lead to poor scar healing. Neither the presence of scar defects nor their measurements were affected by the uterine position.

This study shows that 5.5% of women after a CS manifest no myometrial defects, and presumably, these are the cases with intact uterine anatomy. Therefore, they might have little-to-no increased risk in a subsequent pregnancy assuming that anatomy reflects function. However, the majority of women (94.5%) lose a portion of myometrial thickness at the site of the CS scar with a median RMT ratio of 55.5%. While most studies of CS scars in nonpregnant women deal mainly with absolute measurements of either the niches or the RMT as surrogate markers for adverse outcomes [23], we believe that calculating the RMT ratio is more important, because it represents an individualized value [10]. This line of thinking is not novel, as it has been adopted by other researchers. Seliger et al. measured a deficiency ratio of the niche depth in relation to the combination of RMT and niche depth. Thinning at the scar location with a deficiency ratio greater than 50% was classified as severe deficiency [17]. Our results indicate that such a deficiency is not severe considering that it is very close to our median. If we were to take the 5th percentile as a cut-off point for a severe deficiency ratio, then subjects would need to lose more than 80% of their myometrial thickness before they would be considered as a high-risk group.

We identified two different forms of myometrial deficiencies at the CS scars. The classical type is in the form of a niche where there is a wedge-shaped hypoechogenic defect originating from the uterine cavity (endometrium) and extends with its apex into the myometrium. The prevalence of this form of defect varies between 19 and 69% and this wide range resulted from the lack of a global standard definition [5]. This kind of myometrial defect was present in the majority of women in our cohort at 83.5%. The second form of myometrial defect presents itself as a hyperechogenic dent from the serosa into the myometrium. While we are not the first to describe this finding in post-cesarean uteri, we are among the few who classify it as a myometrial defect. Seliger et al. published a midsagittal transvaginal ultrasound of a post-cesarean uterus showing denting without addressing this finding [17]. Jordans et al.’s guidelines identify this hyperechogenic finding as ‘fibrosis’ that should not me measured as part of RMT [9]. The prevalence of this type of myometrial defect in our cohort is 49.5%. Fibrosis and niche formation are independent of each other and can occur in an isolated or combined fashion. The 94.5% of our cohort that manifested myometrial defects showed isolated fibrosis in 11%, isolated niches in 45% and combined fibrosis and niches in 38.5%. While the medians of the RMT ratios for manifesting either fibrosis or a niche do not significantly differ (around 60%), manifesting fibrosis and niches simultaneously causes a significantly lower RMT ratio of around 40%. This leads us to believe that these patients are at higher obstetrical risk than patients with isolated forms of myometrial defects. Scars with both fibrosis and niches have been described by Fiocchi et al. as retracting scars. Thirty patients examined in their study showed a prevalence of 50% when examined with magnetic resonance imaging (MRI) and 17% when examined with ultrasound [24]. Our prevalence for simultaneous dents and fibrosis is closer to the MRI findings from that study which can be due to improved ultrasound quality compared to the 7 MHz vaginal transducers used in the aforementioned study.

We hypothesize that different adverse outcomes can be attributed to the different scar patterns. Niches represent defects that distort and interrupt the endometrial layer where a subsequent pregnancy could implant. CS pregnancies are regarded as progenitors to PAS [25]. Therefore, patients with exceptionally large niches could be classified as high-risk for PAS. Fibrosis on the other hand might represent interrupted uterine serosa. The uterine wall thins with advancing gestational age as the uterus expands and tension on the myometrium increases. Serosal membranes protect the integrity of the underlying organs [26], and thus, we assume that the uterine serosa protects the integrity of the myometrium by equally distributing the increased tension. A uterus with fibrosis lacks this protection which subsequently increases the tension on the RMT and leads to thinning that results in scar dehiscence and uterine rupture during VBAC.

Our defined measurements were highly reproducible except for the RMT which was affected by fibrosis and niche formation resulting in unreliable variability. The proposed RMT ratio takes these changes into consideration and results in a reproducible quantification with a 0.94 ICC.

The anatomy of the post-cesarean uterus is predominantly anteflexed, and a myometrial loss of about 50% is normally expected. The pattern of this loss is in the form of a predominantly sharp-edged and echogenic niche, fibrosis, or a combination of both. These changes are quantifiable, and their measurements are highly reproducible which can be considered for anticipating adverse outcomes in a subsequent pregnancy. Due to the prospective nature of the BSUM study, the clinical impact of the current data can only be assessed when the outcome of a subsequent pregnancy is acquired. The lack of this information limits the implications of our findings and can be considered as a limitation to our hypothesis in this work.
